# The Effects of Hydrogen-Rich Water on Blood Lipid Profiles in Clinical Populations: A Systematic Review and Meta-Analysis

**DOI:** 10.3390/ph16020142

**Published:** 2023-01-18

**Authors:** Nikola Todorovic, Julen Fernández-Landa, Asier Santibañez, Branislav Kura, Valdemar Stajer, Darinka Korovljev, Sergej M. Ostojic

**Affiliations:** 1 Applied Bioenergetics Lab, Faculty of Sport and Physical Education, University of Novi Sad, 21000 Novi Sad, Serbia; 2 Physical Education and Sports Department, Faculty of Education and Sport, University of the Basque Country (UPV/EHU), 01007 Vitoria, Spain; 3 Centre of Experimental Medicine, Institute for Heart Research, Slovak Academy of Sciences, 841 04 Bratislava, Slovakia; 4 Department of Nutrition and Public Health, University of Agder, Universitetsveien 25, 4604 Kristiansand, Norway; 5 Faculty of Health Sciences, University of Pecs, 7601 Pecs, Hungary

**Keywords:** molecular hydrogen, metabolic syndrome, lipids, triglycerides, hydrogen-rich water

## Abstract

Over the last two decades, a plethora of disease models and human studies have confirmed the beneficial effects of molecular hydrogen (H_2_), a simple biotherapeutic gas. Recent small-scale studies evaluating the effects of hydrogen-rich water (HRW) on various metabolic conditions pointed to advantageous effects of HRW in regulating blood lipid profiles. However, to the best of the authors’ knowledge, no systematic review and/or meta-analysis (SRMA) were published considering HRW consumption and lipid/lipoprotein status. Therefore, the aim of this SRMA was to assess the effects of HRW consumption on blood lipid panel in clinical populations. The search strategy was designed using PRISMA guidelines, and the databases PubMed/Medline, Web of Science, and Scopus were explored from inception until 4 October 2022. A total of seven studies satisfied all the eligibility criteria and were included in SRMA. The results for the pooled meta-analysis showed a significant reduction in total cholesterol, low-density lipoprotein, and triglycerides after HRW intake (*p* = 0.01), with small to moderate effects (pooled SMD = −0.23 (from −0.40 to 0.05); pooled SMD = −0.22 (from −0.39 to 0.04); pooled SMD = −0.38 (from −0.59 to 0.18), respectively). Our findings indicate that drinking HRW can significantly improve lipid status in the clinical populations. Additional studies are warranted to further validate this connection.

## 1. Introduction

Hydrogen is the universe’s oldest, lightest, and most abundant element. Only one electron and one proton form hydrogen, which typically exists as molecular hydrogen in its diatomic form (H_2_). H_2_ has long been regarded as a biologically inert gas with a negligible ability to react with the majority of biomolecules. Although the biotherapeutic effects of H_2_ were reported for the first time almost 50 years ago [1] its use remains far from the mainstream biomedical research. Only recently, with the discovery of the antioxidant properties of H_2_ [2], a large number of disease models and human studies demonstrated the positive effects of additional H_2_ [3]. Actually, over the last two decades, the number of peer-reviewed papers on hydrogen use in experimental and clinical biomedicine exceeded 1000 [4]. These new discoveries drew increasing attention to the use of H_2_, both in general and clinical populations, with hydrogen delivered via various routes and dosage protocols. H_2_ administration strategies typically include drinking hydrogen-rich water (HRW), inhaling H_2_ gas, injecting H_2_-dissolved saline, along with topical administration via H_2_ baths, and buccal consumption of H_2_. Arguably the most popular and convenient way of H_2_ intake is through drinking HRW.

Although the specific molecular target for H_2_ remains unknown, few studies indicated a possible role of this simple medical gas in homeostasis fine-tuning [5,6]. Moreover, research in the past few years revealed additional metabolic roles of hydrogen in humans [7,8]. For instance, H_2_ could stimulate the gene expression of the transcriptional coactivator, peroxisome proliferator-activated receptor 1alpha (PGC-1α), to enhance fatty acid metabolism [9]. HRW is also associated with increased uncoupling protein 1 (UCP1) expression in brown adipose tissue (BAT) [10]. A high concentration of HRW could activate BAT thermogenesis and/or browning of white adipose tissue, potentially leading to increased energy expenditure phenotypes [11]. Finally, H_2_ intervention might increase circulating levels of ghrelin, an obesity-related hormone/neuropeptide that influences appetite [12]. Fasting ghrelin concentrations in patients with simple obesity were lower than in healthy subjects with average body weight [13], whereas H_2_ intervention may improve the regulation of feeding behavior and energy homeostasis. The above pathways might impact several metabolic indices, and perhaps lead to the reduction in total cholesterol (TC) levels and improve lipid status, especially in a population with hypercholesterolemia. It is estimated that ≈ 12% of adults aged 20 and above have abnormal TC levels [14]. High levels of serum TC and low-density lipoprotein (LDL), accompanied by low levels of high-density lipoprotein (HDL), are established risk factors for cardiovascular disease [15,16].

During the last few years, several studies evaluated the effects of HRW on various metabolic conditions, such as non-alcoholic fatty liver disease (NAFLD) [17,18], obesity, or metabolic syndrome [12,19], pointing to possible effects of HRW in regulating lipoprotein and lipid status. However, to the best of the authors’ knowledge, there is no systematic review and meta-analysis (SRMA) published considering H_2_ consumption and lipid status. Therefore, this SRMA aims to assess the effects of HRW consumption on blood lipid profiles in various clinical populations.

## 2. Results

### 2.1. Literature Search

**Figure 1:**
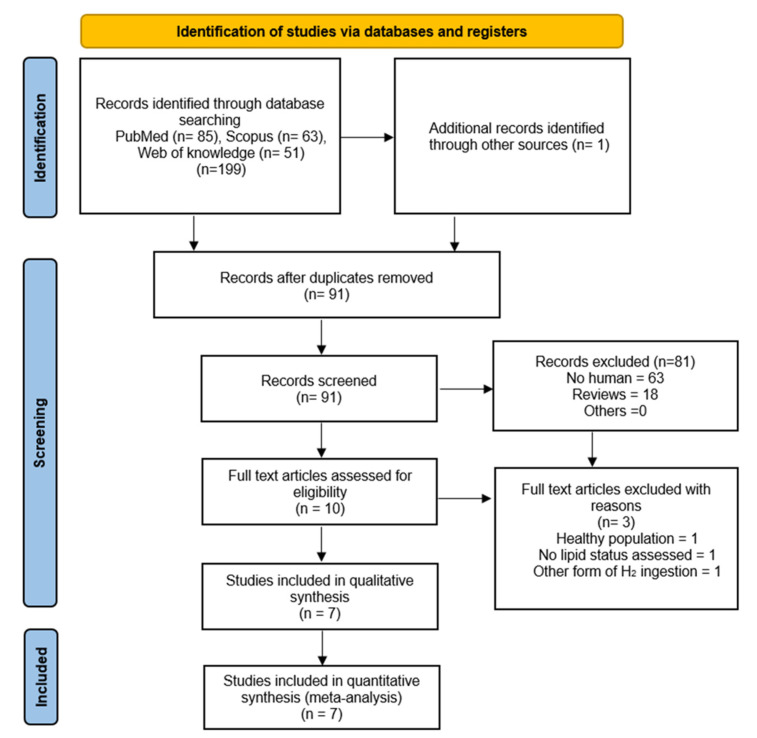
Prisma flow diagram.

A total of 199 records were found through the database, including 90 unique records and 108 duplicates, while one study [20] was identified through the snowball strategy. After 81 studies with no relevance were removed through title and abstract screening, ten studies were eligible for full-text screening. Finally, seven articles were considered to be included in this SRMA, involving 256 participants [12,17,18,19,20,21,22]. The study search was conducted by 4 October 2022. The PRISMA flow diagram is depicted in Figure 1.

All relevant information regarding the studies that meet the inclusion criteria is presented in Table 1. The study population contained participants with various conditions. In two studies [17,18], participants were older adults with NAFLD, two studies evaluated overweight and/or patients with hypercholesterolemia [12,21], while the remaining three studies evaluated patients with T2DM [22], metabolic syndrome [21], or elderly population with accompanying diseases [20].

In terms of intake, all participants in the studies ingested HRW. Participants in five of the seven studies consumed approximately 1 L per day of HRW in three separate dosages [17,18,19,21,22]. In one study [20], participants were given 500 mL/d of HRW divided into two doses, whereas in one study the amount of HRW consumed was not reported [12]. The duration of the studies ranged from four to 24 weeks [12,19,22].

In six of seven analyzed studies, lipid profiles were assessed through the following blood analysis: TC, HDL, LDL, and triglycerides [12,17,18,19,20,22]. Only the study by Song et al. [21] evaluated TC, HDL, and LDL, with the exception of triglycerides.

Two studies reported a significant reduction in TC levels after HRW intake [19,22]. Five studies reported significant decrease in LDL test outcomes [18,19,20,21,22], while one study reported an increase in HDL levels [22]. Finally, the triglyceride levels were significantly decreased in two studies [12,19].

### 2.2. Level of the Quality of the Studies

The PEDro scale mean score for the included studies was 9.28, considered as excellent quality. Details considering PEDro scale were explained elsewhere [23]. Six studies [12,17,18,19,22] were classified as excellent quality and one investigation [20] was categorized as good quality. The results of the PEDro scale are presented in Table 2.

### 2.3. Pooled Effect Estimate

**Figure 2:**
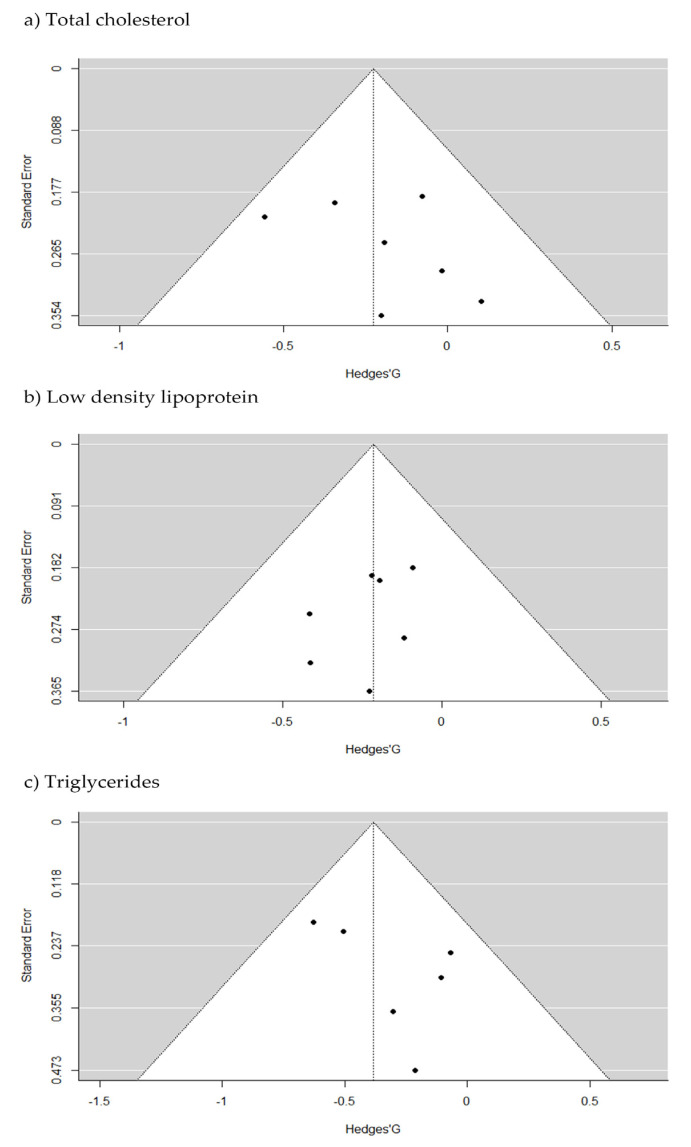
Funnel plots of included studies. Funnel plot (standard error vs. Hedges’ G) for all of the included studies in this SRMA. There were no risks of heterogeneity considering total cholesterol (I^2^ = 0%; *p* = 0.49), low density lipoprotein (I^2^ = 0%; *p* = 0.95) and triglycerides (I^2^ = 0%; *p* = 0.47).

In this SRMA, we conducted several data sub-analyzes. The I^2^ square test noticed no significant heterogeneity between studies for total cholesterol (*p* = 0.49), triglycerides (*p* = 0.47), and LDL (*p* = 0.95). Further, the I^2^ statistic observed a no risk of heterogeneity (I^2^ = 0%) in all sub analysis. The visual analysis of the funnel plot did not indicated asymmetry showing publication bias (Figure 2). Moreover, no significant results were found in the Egger’s regression test for funnel plot asymmetry (df = 5; *p* = 0.39), (df = 5; *p* = 0.21), (df = 5; *p* = 0.28), respectively.

**Figure 3:**
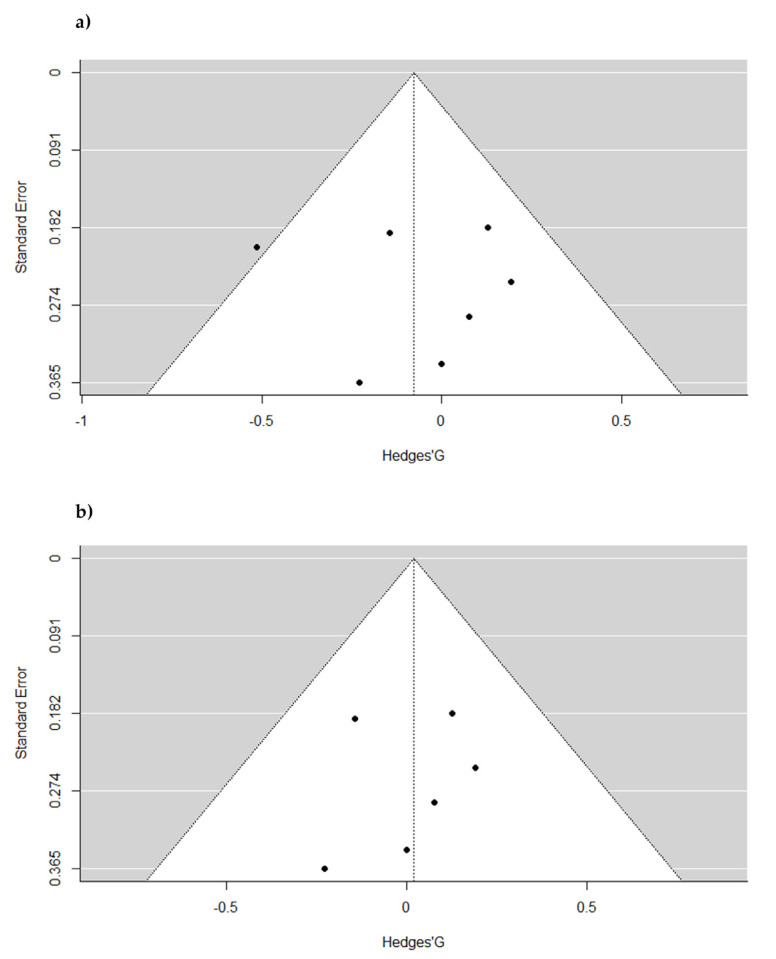
Funnel plots of included studies considering High-density lipoproteins. Funnel plot (standard error vs. Hedges’ G). (**a**) represent all of the studies included in this SRMA. (**b**) represent the results after excluding one study not evenly distributed around the base of the funnel plot. After the exclusion of one study, there were no risks of heterogeneity (I^2^ = 0%; *p* = 0.84).

For the HDL levels the I^2^ square test noticed no significant heterogeneity between studies (*p* = 0.12). Nevertheless, the I^2^ statistic observed a moderate risk of heterogeneity (I^2^ = 20.85%). The visual analysis of the funnel plot indicated asymmetry showing publication bias (Figure 3a). However, no significant results were found in the Egger´s regression test for funnel plot asymmetry (df = 5; *p* = 0.86), and Duval and Tweedie Trim and Fill´s method did identify one missing studies on left side of the plot. After excluding the studies not evenly distributed around the base of the funnel plot, the heterogeneity between studies was drastically reduced, showing a low risk of heterogeneity (I^2^ = 0%; *p* = 0.84). Egger´s regression test showed no funnel plot asymmetry (df = 5; *p* = 0.84) and Duval and Tweedie Trim and Fill´s method did not identify missing studies on either side of the plot after the bias correction. Funnel plots are displayed in Figure 3b.

**Figure 4:**
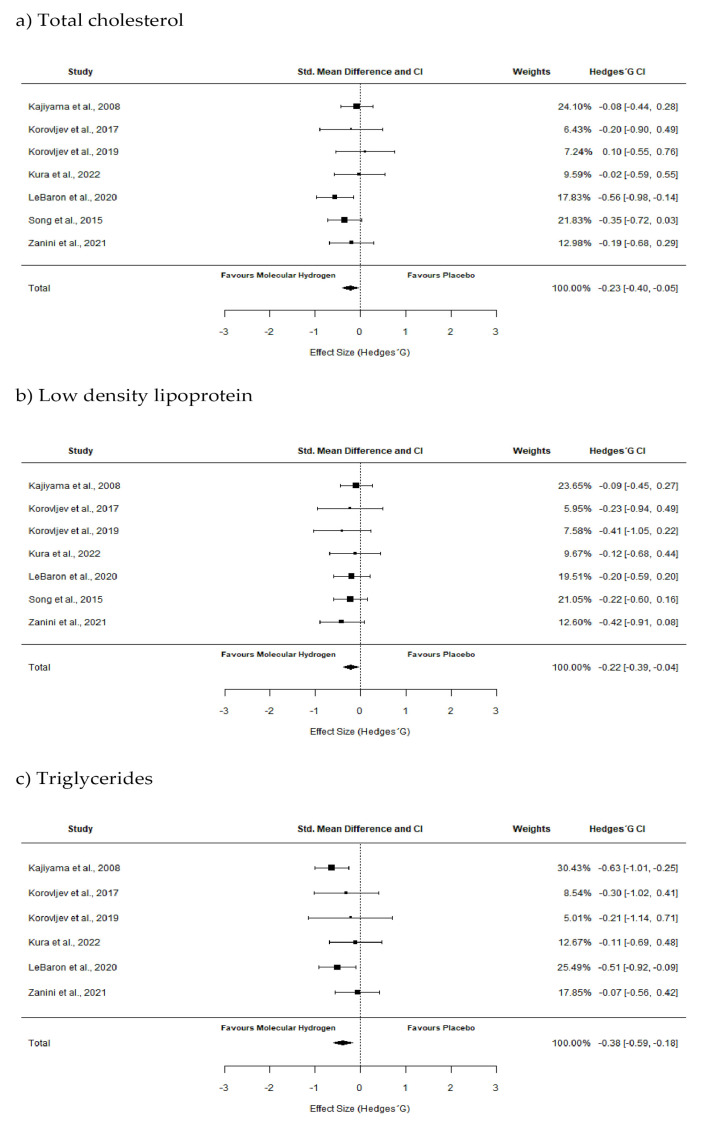
Forest plots of included studies. Forest plot comparing the effects of HRW supplementation on: (**a**) total cholesterol with a small negative effect (pooled SMD = −0.23 [from −0.40 to 0.05]; *p* ≤ 0.01), (**b**) low-density lipoprotein with small effect (pooled SMD = −0.22 [from −0.39 to 0.04]; *p* ≤ 0.01), and (**c**) triglycerides, with a moderate negative effect (pooled SMD = −0.38 [from −0.59 to 0.18], *p* ≤ 0.01).

The results of the pooled meta-analysis showed significant change on total cholesterol levels after HRW supplementation in clinical population (*p* ≤ 0.01), with a small negative effect (pooled SMD = −0.23 (from −0.40 to 0.05)). Regarding the triglycerides levels, the result showed significant decrease after HRW supplementation (*p* ≤ 0.01), with a moderate negative effect (pooled SMD = −0.38 (from −0.59 to 0.18)). Considering LDL, HRW supplementation lowers LDL levels (*p* = 0.02) with small effect (pooled SMD = −0.22 (from −0.39 to 0.04)). Forest plots are shown in Figure 4.

**Figure 5:**
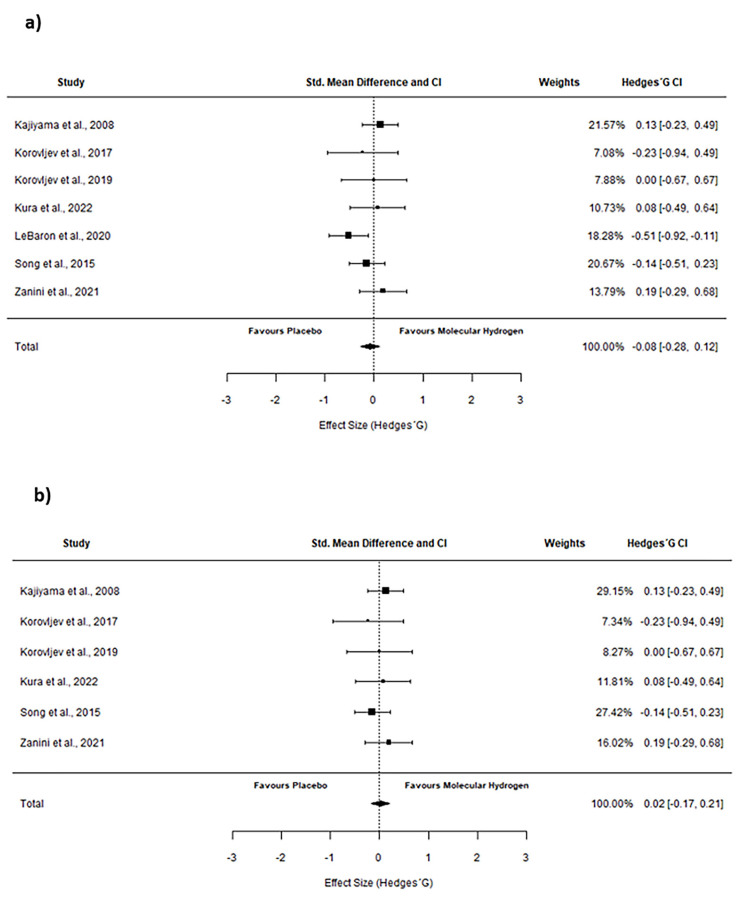
Forest plots of included studies considering High-density lipoproteins. Forest plot comparing the effects of HRW supplementation on HDL. (**a**) represent results from all of the studies included in this SRMA. (**b**) represent the results after excluding one study not evenly distributed around the base of the funnel plot. The results for all studies showed a trivial negative effect (pooled SMD = −0.08 [from −0.28 to 0.12], *p* = 0.23); Following the exclusion of not evenly distributed study, the results were similar (pooled SMD = 0.02 [from −0.17 to 0.21; *p* = 0.83).

Only for HDL analysis, the results for the pooled meta-analysis showed a non-significant change after HRW supplementation (*p* = 0.23), with a trivial negative effect (pooled SMD = −0.08 (from −0.28 to 0.12)) (Figure 5a). Following the exclusion of the studies not evenly distributed around the base of the funnel plot, the results were similar (pooled SMD = 0.02 (from −0.17 to 0.21; *p* = 0.83)). Forest plots are shown in Figure 5b.

## 3. Discussion

The main purpose of this SRMA was to analyze and summarize the current scientific literature in order to assess the efficacy of HRW consumption on blood lipoprotein and lipid status in clinical populations. Seven studies fulfilled the inclusion criteria, and a total of 279 participants took part in the analysis. The primary finding of this SRMA was that HRW intake had significant positive effects for decreasing serum TC, LDL, and triglycerides, while there were no changes in HDL levels.

Two studies evaluated the effects of HRW on NAFLD [17,18]. NAFLD affects approximately 25% of the global population, making it the most common hepatic pathology [24]. Interestingly, the results from these two studies indicated only a small and non-significant influence of HRW on lipid status. Certain positive trends were observed in study by Kura et al. [17], however most of them did not reach a statistical significance. On the other hand, in a study by Korovljev et al. [18], the administration of HRW markedly reduced liver fat content (~20%) but did not change serum levels of lipid status, indicating that liver may be a target of HRW. The study by Ichihara et al. [25] revealed that upon H_2_ intake, the concentration of H_2_ is elevated in the blood for 1 to 2 h afterward. However, H_2_ has not been detected in the carotid artery, and it is possible that H_2_ was subsequently metabolized in the liver or eliminated via the lungs. This finding can be explained due to the fact that the liver could be a target tissue by oral HRW intake, or because H_2_ works firstly in the liver and reduces liver fat, and then affects serum levels of lipids. Second, the lack of HRW effects on metabolic profiles in NAFLD might be due to a lower dosage of hydrogen ingestion, with previous trials reported usage of ~6 ppm of H_2_ per day [12] and/or short period of supplementation duration. Additionally, the H_2_ concentration in HRW in studies included here ranges from 4.76 ppm [18] to 15 ppm [20]. Moreover, it might be possible, that patients with NAFLD may not have the benefit of drinking HRW in terms of reduction of total cholesterol and triglycerides, while future studies should evaluate primary targets of HRW ingestion in this specific population.

In all of the other included studies, decreased levels of TC and/or LDL and/or triglycerides were observed, while the levels of HDL mainly were unchanged [12,19,20,21,22]. This promising result indicates a possible role in HRW among the population that had certain problems considering the regulation of lipoprotein and lipid status. The effects of H_2_ even today are highly debatable, but it seems that H_2_ has more effects in the population that had specific conditions, such as obesity, metabolic syndrome, and diabetes, compared to a healthy population. There are few mechanisms that could explain this. HRW has the potential to influence PGC-1α activation [9]. PGC-1a is a transcription coactivator that facilitates mitochondrial biogenesis, which is essential for controlling cellular energy metabolism [26]. Furthermore, PGC-1α raises hepatic and circulating levels of fibroblast growth factor 21 (FGF-21). Improved insulin sensitivity via induction of hepatic FGF-21 is one possible mechanism by which HRW regulates lipid metabolism, resulting in better fuel utilization and less body fat accumulation [27]. Moreover, FGF-21 can also efficiently regulate metabolic homeostasis and cellular aging. Simultaneously, FGF-21 analogs and FGF-21 receptor agonists show promise as a treatment approach for aging-related metabolic diseases [28]. PGC-1α protein accumulation has also shown increases in uncoupled respiration in BAT. Previously, HRW was linked to an increase in UCP1 expression in the BAT of high-fat diet-induced obesity mice [10]. UCP1 is an important thermogenic factor in BAT mitochondria. Supplemental HRW’s thermogenic effects could augment traditional obesity treatment, with HRW-mediated UCP1 upregulation stimulating whole-body energy expenditure and possibly regulating lipid status. [11].

Another possible role of H_2_ can be the regulation of ghrelin hormone. Ghrelin has recently been identified as an important modulator of mitochondrial bioenergetics [29]. H_2_ activation of ghrelin receptors (GHS-R1) could set off a chain reaction of energy-related pathways [29]. When exposed to high ghrelin concentrations, human Sertoli cells increase protein levels of complexes III and V, which are key carrier proteins for extracting energy in the mitochondrial electron transport chain [30]. Furthermore, ghrelin levels were found to be significantly decreased in obese patients, indicating that an H_2_ increase in ghrelin secretion may regulate energy metabolism and feeding behaviors and thus affect lipid metabolism.

Finally, H_2_ can affect obesity by decreasing oxidative stress and inflammation in adipose tissues. Adipocytes with dysfunctional mitochondria are less responsive to energy utilization and more susceptible to apoptosis and oxidative stress, resulting in fat accumulation, inflammation, and obesity-related pathologies [31]. Few papers reported that HRW could influence decreased lipid peroxidation. However, this was followed by increasing levels of malondialdehyde (MDA). For example, in the study by Kura et al. [17], a mild non-significant increase in MDA (~17.2%) was detected. Similar trends were also observed in other studies [19,20], while these values are often elevated in pathological conditions. Lower lipid peroxidation may protect cellular membranes from oxidative degeneration and possibly affect metabolism regulation [32].

Although the effects of H_2_ have been shown in numerous studies, H_2_ impact is often questionable, mainly because of its debatable selective antioxidant capability. Due to the limited H_2_ solubility (about 1.6 mL/100 mL in water and 3.0 mL/100 mL in fat), the selective antioxidant activity of this molecule is difficult to explain [33]. At such a low concentration and relatively low reducing power, it is difficult for H_2_ to react directly with other chemical components that are weakly oxidizing. Regarding reaction rate, the reaction between hydroxyl radical and other biological molecules is 1000 times quicker than the reaction between hydroxyl radical and H_2_ [33]. Moreover, the theoretical models present in this study omit these antioxidant properties. In addition, this study only proposes a possible mechanism and explanations of the H_2_ effects on lipid metabolism, while the direct mechanism of H_2_ is still unknown. Nevertheless, the beneficial effect of H_2_ was shown in more than 500 studies in various disease models and certainly deserves to be researched more in the future to confirm the present and discover new findings.

Our study has some limitations inherited from all meta-analyses [34], but the use of rigorous inclusion criteria, and double-blind RCTs, limited the publication bias. However, this led to a small number of studies being included in this SRMA, which can impact the strength of observed results and conclusions. Second, the following factors varied among studies: baseline characteristics of participants, supplementation intake protocols, duration of studies, accompanying diseases, and possible interference with regular therapies that participants received, indicating a possible risk of bias. However, an Egger test of all the included studies showed that the bias was insignificant. Third, we could not confirm the long-term effects of HRW due to the short follow-up periods (ranging from four weeks to six months) in the included studies. Finally, although we presented some of the mechanisms that can affect lipid metabolism, the direct actions of HRW still need to be discovered. Consequently, well-designed and adequately powered large-scale RCTs are needed to raise the level of evidence.

## 4. Materials and Methods

The PRISMA^®^ (Preferred Reporting Items for Systematic Reviews and Meta-Analyses) statement guidelines [35] were followed to assess HRW supplementation’s effects on the clinical population’s lipid metabolism. The study protocol was registered in the PROSPERO (Prospective Register of Systematic Reviews) with the confirmatory number CRD42022364471.

PubMed, Web of Science, and Scopus databases were searched using a systematic approach for relevant articles published before 4 October 2022. The principal author (N.T.) carried out the search independently, and any doubts were solved by consultation with another author (SMO). To find records, the following Boolean search terms were used in different combinations: (“molecular hydrogen” OR “dihydrogen” OR “Hydrogen rich water” OR “HRW supplementation “) AND (“lipid metabolism” AND obesity) AND (NAFLD OR Diabetes OR DMT2). In addition, the references of the detected articles were manually inspected using snowball strategy [36] to find articles that were not identified in the initial search.

### 4.1. Inclusion and Exclusion Criteria

Review articles and unpublished abstracts, theses, and dissertations were not considered for inclusion. Only human experimental trials were considered for inclusion. Furthermore, the following inclusion criteria were used to choose the articles for this SRMA: (i) HRW /intake; (ii) clinical population (metabolic diseases and/or obesity and/or NADLD and/or Diabetes); (iii) HRW supplementation effects involving the following tests: total cholesterol, high-density lipoprotein, low-density lipoprotein, triglycerides; (iv) human experimental trial; (v) controlled with a placebo group; (vi) original and peer-reviewed studies written in English.

On the contrary, studies were excluded when: (i) other forms of H_2_ supplementation (e.g., inhalation, topic ingestion, saline injection, eye drops, buccal administration) (ii) HRW was combined with other supplements; (iii) participants of the studies were not considered as a clinical population; (iii) a lack of placebo group for the comparison of the results; (iv) studies that do not include pre- and post-supplementation data.

### 4.2. Text Screening

All titles and abstracts were assessed for eligibility by the main author (N.T.) and were also independently screened by two researchers (J.F.-L. and A.S.), with uncertainty regarding eligibility discussed among the researchers in order to reduce the number of studies that did not meet the inclusion and exclusion criteria. The full texts were then screened by the same researchers to determine which experimental trials should be included in the SRMA.

### 4.3. Data Extraction and Study Coding

Whenever available, the following data were extracted from each study that met the inclusion criteria: study authors and publication year, study design, basic participant information, supplementation protocol, duration of supplementation protocol and blood lipids outcomes (e.g., pre- and post-data). When values were plotted as figures, the authors were directly contacted in order to provide raw data. The relevant study characteristics from each study that met the inclusion criteria were carefully reviewed and tabulated in a spreadsheet (Microsoft Excel, Microsoft Corporation, Washington, DC, USA).

In case that some of the included studies in this SRMA reported more than one outcome measuring lipoprotein and lipid status, the “MAd” package in R software (R Foundation for Statistical Computing, Vienna, Austria) [37] was used to compute a single, aggregated effect size estimate for each study in the event [38]. A generalized estimate of the within-study correlation between the relevant variables was calculated using the mean of these correlation coefficients (r = 0.70) [23].

### 4.4. Quality Assessment of Included Studies

PEDro scale was assessed to evaluate the methodological quality of included studies [39]. The process was conducted by researchers (N.T. and A.S.), while potential reviewer discrepancies were resolved through discussion. This tool is constructed of 11 different items. Items 2 to 11 can be rated with a 0 or a 1, so the highest possible rate on the PEDro scale is 10 (low risk of bias), and the lowest 0 (high risk of bias). Further studies with scores (0–3 points) are considered as poor quality (4–5 points), fair quality (6–8 points), good quality and excellent quality (when the score was 9–10 points).

### 4.5. Statistical Analysis

The R programming language was used to perform a meta-analysis using a random effects modeling strategy. The inverse variance method was used to pool the weighted estimation of standardized mean differences (SMD) across studies. The SMD was used to calculate the magnitude of the effect, with 0.2 indicating trivial, 0.2–0.3 small, 0.4–0.8 moderate, and >0.8 denoting large effect [40]. The I^2^ statistic [41] was used to assess statistical heterogeneity across different trials in the meta-analysis, where 25% indicates low risk of heterogeneity, 25–75% means moderate risk of heterogeneity, and >75% indicates significant risk of heterogeneity [41]. The restricted maximum-likelihood estimator of 2 was used to compute the I^2^ statistic. Plotting standard errors against Hedges’ G values for the included studies made it possible to evaluate any potential funnel plot asymmetry visually [42]. Egger’s regression test [43] the Trim and Fill method of Duval and Tweedie [44] was used to assess funnel plot asymmetry additionally.

## 5. Conclusions

In conclusion, the current SRMA shows that HRW intake significantly improves several elements of blood lipid profiles in diverse clinical populations. HRW likely affects cellular bioenergetics and lipid metabolism via several pathways and plays a role in cellular fine-tuning. However, future studies should comprehend fundamental research evaluating the direct effect of H_2_ on energy metabolism to understand better how H_2_ influences homeostasis.

## Figures and Tables

**Table 1:** Effects of molecular hydrogen on lipid metabolism.

Study	Population	Intervention	Variables Analyzed	Outcomes
Kajiyama et al., 2008 [22]	36 subjects with T2DM (58.6 ± 4.7 y)	*Study type:* Randomized, double-blind, placebo-controlled crossover study *Supplementation protocol:* 900 mL/d of HRW (H_2_ concentration = 12 ppm) or placebo water *Duration:* 8 weeks	TC LDL sdLDL HDL TG	↔ ↔ ↓ ↑ ↔
Song et al., 2015 [21]	68 subjects (35–60 years old) with hypercholesterolemia	*Study type:* Randomized, double-blind, placebo-controlled study *Supplementation protocol:* 0.9 L/d (0.3 L 3 times/d) of HRW (H_2_ concentration ≈ 0.6 ppm) or placebo *Duration:* 12 weeks	TC LDL HDL	↓ ↓ ↔
Korovljev et al., 2018 [12]	10 middle-aged overweight women (56.4 ± 12.6 y)	*Study type:* Randomized, double-blind, placebo-controlled crossover study *Supplementation protocol:* blend of hydrogen generating minerals (46 mg of calcium and 40 mg of magnesium) (H_2_ concentration ≈ 6 ppm) or placebo (cellulose) dissolved in water *Duration:* 4 weeks	TC LDL HDL TG	↔ ↔ ↔ ↓
Korovljev et al., 2019 [18]	12 subjects with NAFLD (56.2 ± 10 y)	*Study type:* Randomized, double-blind, placebo-controlled study *Supplementation protocol:* 4 doses daily of 250 mL HRW (H_2_ concentratio = 4.76 ppm) or placebo water *Duration:* 4 weeks	TC LDL HDL TG	↔ ↓ ↔ ↔
LeBaron et al., 2020 [19]	60 subjects with metabolic syndrome(43.4 ± 9.2 y)	*Study type:* Randomized, double-blind, placebo-controlled study *Supplementation protocol:* 3 doses daily of 250 mL HRW (H_2_ concentration > 5.5 ppm) or placebo water *Duration:* 24 weeks	TC LDL VLDL HDL TG	↓ ↓ ↓ ↔ ↓
Zanini et al., 2021 [20]	40 elderly man and women (76 ± 5.6 y)	*Study type:* Randomized, double-blind, placebo-controlled study *Supplementation protocol:* 500 mL/d of HRW (H_2_ concentratio = 15 ppm) or Placebo water *Duration: 24 weeks*	TC LDL HDL TG	↔ ↓ ↔ ↔
Kura et al., 2022 [17]	30 subjects with NAFLD (53.2 ± 9.1 y)	*Study type:* Randomized, double-blind, placebo-controlled study *Supplementation protocol:* 3 doses daily of 330 mL HRW (H_2_ concentratio > 4 ppm) or placebo water *Duration:* 8 weeks	TC LDL HDL TG	↔ ↔ ↔ ↔

**Abbreviations:** ↔—no change; ↑—statically significant increase; ↓—statically significant decrease; TC—total cholesterol; LDL—low density lipoprotein; sdLDL—small-danse low density lipoprotein; HDL—high density lipoprotein; TG—triglycerides; VLDL—very low density lipoprotein; NAFLD—non-alcoholic fatty liver disease; T2DM—diabetes mellitus type 2; HRW—hydrogen-rich water; ppm—parts per million.

**Table 2:** Table PEDro ratings of the included studies.

Study	1	2	3	4	5	6	7	8	9	10	11	TOTAL
Kajiyama et al., 2008	Yes	1	0	1	1	1	1	1	1	1	1	9
Song et al., 2015	Yes	1	1	1	1	1	1	1	1	1	1	10
Korovljev et al., 2018	Yes	1	0	1	1	1	1	1	1	1	1	9
Korovljev et al., 2019	Yes	1	0	1	1	1	1	1	1	1	1	9
LeBaron et al., 2020	Yes	1	1	1	1	1	1	1	1	1	1	10
Zanini et al., 2021	Yes	1	0	1	1	0	1	1	1	1	1	8
Kura et al., 2022	Yes	1	1	1	1	1	1	1	1	1	1	10

## Data Availability

Data sharing not applicable.
